# Antibacterial Albumin-Tannic Acid Coatings for Scaffold-Guided Breast Reconstruction

**DOI:** 10.3389/fbioe.2021.638577

**Published:** 2021-03-31

**Authors:** Silvia Cometta, Nathalie Bock, Sinduja Suresh, Tim R. Dargaville, Dietmar W. Hutmacher

**Affiliations:** ^1^Centre in Regenerative Medicine, Institute of Health and Biomedical Innovation, Queensland University of Technology, Brisbane, QLD, Australia; ^2^School of Mechanical, Medical and Process Engineering, Science and Engineering Faculty, Queensland University of Technology, Brisbane, QLD, Australia; ^3^Translational Research Institute, Brisbane, QLD, Australia; ^4^School of Biomedical Sciences, Faculty of Health, Queensland University of Technology, Brisbane, QLD, Australia; ^5^ARC Industrial Transformation Training Centre for Multiscale 3D Imaging, Modelling and Manufacturing, Queensland University of Technology, Brisbane, QLD, Australia; ^6^School of Chemistry and Physics, Science and Engineering Faculty, Queensland University of Technology, Brisbane, QLD, Australia; ^7^ARC Industrial Transformation Training Centre in Additive Biomanufacturing, Queensland University of Technology, Brisbane, QLD, Australia

**Keywords:** bacterial infection, antibacterial coating, polycaprolactone, 3D printing, scaffold, albumin, tannic acid

## Abstract

Infection is the major cause of morbidity after breast implant surgery. Biodegradable medical-grade polycaprolactone (mPCL) scaffolds designed and rooted in evidence-based research offer a promising alternative to overcome the limitations of routinely used silicone implants for breast reconstruction. Nevertheless, as with any implant, biodegradable scaffolds are susceptible to bacterial infection too, especially as bacteria can rapidly colonize the biomaterial surface and form biofilms. Biofilm-related infections are notoriously challenging to treat and can lead to chronic infection and persisting inflammation of surrounding tissue. To date, no clinical solution that allows to efficiently prevent bacterial infection while promoting correct implant integration, has been developed. In this study, we demonstrated for the first time, to our knowledge that the physical immobilization of 1 and 5% human serum albumin (HSA) onto the surface of 3D printed macro- and microporous mPCL scaffolds, resulted in a reduction of *Staphylococcus aureus* colonization by 71.7 ± 13.6% and 54.3 ± 12.8%, respectively. Notably, when treatment of scaffolds with HSA was followed by tannic acid (TA) crosslinking/stabilization, uniform and stable coatings with improved antibacterial activity were obtained. The HSA/TA-coated scaffolds were shown to be stable when incubated at physiological conditions in cell culture media for 7 days. Moreover, they were capable of inhibiting the growth of *S. aureus* and *Pseudomonas aeruginosa*, two most commonly found bacteria in breast implant infections. Most importantly, 1%HSA/10%TA- and 5%HSA/1%TA-coated scaffolds were able to reduce *S. aureus* colonization on the mPCL surface, by 99.8 ± 0.1% and 98.8 ± 0.6%, respectively, in comparison to the non-coated control specimens. This system offers a new biomaterial strategy to effectively translate the prevention of biofilm-related infections on implant surfaces without relying on the use of prophylactic antibiotic treatment.

## Introduction

Current post-mastectomy breast reconstruction options such as autologous tissue flaps or silicone-based implants are limited and present high rates of local complications including capsular contracture, infection and seroma formation (Visscher et al., 2017). Scaffold-guided tissue engineering (SGTE) represents a promising alternative to breast reconstruction by promoting tissue regeneration through the use of patient-specific implants with highly controlled micro and macro-scale morphology (Hutmacher et al., 2007). Nevertheless, as with any implant, scaffolds are highly susceptible to infection, due to the surgically induced immune response at the interface between the biomaterial and the host tissue (Zimmerli and Trampuz, 2013; Riool et al., 2017).

Biomaterial-related infections implicate numerous sophisticated interactions between bacteria and the implant surface, as well as the host reaction to both. Breast implants are placed in a disinfected but not sterile environment. Even if bacteria contamination from the skin is minimized, all or part of the breast implant directly abuts the breast parenchyma itself, which is colonized by a multitude of bacterial species including *Propionibacterium* and *Staphylococcus* spp. Once an implant is surgically introduced into the body, the biomaterial interfacing with the surgically created large wound stimulates a local host response. This involves acute and chronic inflammation, development of granulation tissue, foreign body reaction and, in the worst case scenario, a clinically detected fibrous encapsulation (Anderson et al., 2008). This results in an immuno-incompetent, pro-inflammatory microenvironment, which increments the vulnerability of the biomaterial to bacterial infection (Schierholz and Beuth, 2001; Arciola et al., 2018). The first step in biomaterial-associated infections is bacteria adhesion to the surface. The bacteria then aggregate and start to produce extracellular polymeric substance (EPS), which plays a key role in nutrition, protection and anti-microbial resistance (Brileya et al., 2014). This assemblage of microbial cells and the EPS is defined as biofilm and once in this state, bacteria are significantly more tolerant to antimicrobials and less accessible to the host immune defense system in comparison to their planktonic counterparts (Riool et al., 2017). The role of biofilm formation in breast implant-related complications has been demonstrated in humans and modeled in large animal models (Pajkos et al., 2003; Tamboto et al., 2010; Silvestri et al., 2011). Coagulase-negative staphylococci have been described as the predominant bacteria in breast implant biofilm formation–*Staphylococcus aureus*, in particular. Biofilm formation has been directly implicated in the pathogenesis of both capsular contracture and breast implant-associated anaplastic large cell lymphoma (BIA-ALCL) (Virden et al., 1992; Pajkos et al., 2003). Fibrous capsule formation is a normal response to foreign bodies such as silicone breast implants. This process becomes pathologic when contracture occurs, leading to deformity and pain. Conversely, capsular contracture is not a normal response and its cause is likely multifactorial. One of the leading hypotheses is induction of an overactive and persistent inflammatory process from a biofilm formed on the implants (Adams, 2009). This leads to constant recruitment of cells such as macrophages, fibroblasts and myofibroblasts to the implant surface and eventually contracture (Ajdic et al., 2016).

A more recent complication of breast implants is BIA-ALCL. It is a relatively new clinicopathologic entity which is yet to be fully understood, with a connection between silicone breast implants and large cell lymphoma first identified in 2011 (Ebner et al., 2019). It has been shown to be intimately related to the formation of biofilm on breast implants (Kadin et al., 2016). The mechanism of this is related to T-cell stimulation from antigens that become aberrant and malfunction in some patients. To date, documented cases of BIA-ALCL have only occurred on textured silicone implants (Clemens et al., 2019). The suspected link between textured silicone implants and BIA-ALCL is the increased surface area and therefore an increased chance of harboring bacteria. The emergence of BIA-ALCL has heightened awareness of the role of bacteria and biofilm formation in both BIA-ALCL and capsular contracture. As a result, there is an increasing need for local treatment options that can potentially prevent subclinical infection, as well as maintain breast contour. Current prophylactic measures used to prevent breast implant infections, involve the administration of high-dose systemic antibiotics perioperatively. However, systemic administration of antibiotics frequently fails as a result of the poor concentration of antibiotics reaching the implant site. In addition, the rapid emergence of multidrug-resistant bacteria has further impaired the effectiveness of antibiotic therapy (Riool et al., 2017). As an example, 68% of *S. aureus* isolated from breast implant infections are found resistant to methicillin, a broad-spectrum antibiotic (Prasad et al., 2019).

Novel alternatives to combat bacterial adhesion and biofilm formation include tailoring the antibacterial properties of the biomaterial surface. In particular, the development of multiple-function surface modifications and coatings that not only allow incorporating new alternative antimicrobial agents with improved spectrum of activity, but also promote tissue integration and favorable host response, is a promising alternative to achieve reduced infection and healing times, and consequently, a better performance of biomaterial implants. As an example, our group has previously reported the fabrication of tissue engineered scaffolds with pre-designed macro- and microporosity, through the use of fused deposition modeling (FDM) and the porogen leaching technique. The micro-scale porosity conferred the scaffolds with a drug delivery functionality by facilitating the physical immobilization and release of therapeutic agents, while the large interconnected pores promoted the remodeling and regeneration of large volumes of adipose tissue (Chhaya et al., 2016; Dang et al., 2019).

Human serum albumin (HSA) is a biodegradable and non-immunogenic protein that has long been studied as a coating component for the surface modification of biomaterials to minimize non-specific protein adsorption, improve cell adhesion and inhibit bacteria attachment to the surface of implants (Tao et al., 2019). For instance, Horváthy et al. (2019) showed that albumin is able to induce endogenous progenitor recruitment, promoting host cell attachment and proliferation for the support of bone remodeling, when coated onto bone grafts. In addition, several studies have demonstrated the ability of albumin coatings to inhibit gram-positive and gram-negative bacteria adhesion onto different material surfaces such as polymers and metals (Kinnari et al., 2005; Ruiz et al., 2011). The antibacterial properties of albumin have been attributed to its ability to bind to bacterial cells and change some surface properties such as the surface energy (Ribeiro et al., 2012). Other studies suggest that albumin is able to bind and sequester homoserine lactone quorum signals molecules secreted by *Pseudomonas aeruginosa* during quorum sensing, a mechanism by which bacteria moderate vital processes such as bacterial growth, adhesion and biofilm formation (Smith et al., 2017). Due to the ability of albumin to promote tissue integration while decreasing bacteria colonization, albumin coatings have been studied on different biomedical devices such as tympanostomy tubes, cochlear implants and urinary implants (Horváthy et al., 2017). Nevertheless, to the best of our knowledge, no studies have been performed on resorbable materials, which are fundamental for tissue engineering applications. Likewise, no studies have reported synergistic antibacterial effects of albumin coatings in combination with other antimicrobial agents.

Tannic acid (TA) is an FDA-approved flavoring and adjuvant agent for food, with known multifunctional properties, including antioxidant, antibacterial, and antimutagenic characteristics (Chen et al., 2003; Gülçin et al., 2010; Aguilera et al., 2016). In particular, TA has shown to be able to inhibit biofilm formation by downregulating the expression of quorum sensing genes in gram-negative bacteria (Trentin et al., 2013; Chang et al., 2014; Mangwani et al., 2015), as well as by causing an increase in extracellular lytic transglycolase IsaA levels, which play a key role in the synthesis and degradation of peptidoglycans, in gram-positive bacteria (Payne et al., 2013). In addition to these properties, TA has the ability to form complexes or cross-link macromolecules at several binding sites via different interactions, such as hydrogen bonding as well as hydrophobic and electrostatic interactions (Fan et al., 2017).

In this study, we developed a stable and effective antibacterial coating for 3D printed mPCL scaffolds with a dual macro- and microporous architecture, by immobilizing HSA and TA on the biomaterial surface. For this purpose, HSA was first immobilized onto the surface of the scaffolds through adsorption and ionic forces. HSA-coated scaffolds were then treated with a TA solution. Once TA and HSA molecules encounter; they form a stable complex through hydrogen-bonding and hydrophobic interactions, allowing HSA-stabilization/crosslinking as well as TA immobilization on the scaffold surface. Herein, we characterize the microporosity of the fabricated scaffolds, as well its influence on the mechanical properties of clinically relevant large scaffolds. Moreover, we investigate the stability of the newly developed antibacterial coating and its efficacy against two of the most commonly found bacteria in breast implant-infections, *S. aureus* and *P. aeruginosa*.

## Materials and Methods

### Materials

Lyophilized HSA (≥96%), TA (ACS grade), fluorescein 5(6)-isothiocyanate (FITC), and sucrose (≥99.5%) were purchased from Sigma-Aldrich (United States). Dulbecco’s Modified Eagle Medium (DMEM) formulated with 4.5 g/L D-Glucose, L-Glutamine and fetal bovine serum (FBS) were obtained from ThermoFisher Scientific (United States), mPCL (MW: 50,000) was purchased from 3D4Makers (Netherlands).

### Methods

#### Scaffold Design and Fabrication

Macroporous lattice scaffolds of a medical grade PCL composite containing 45% (w/w) of sugar particles with crystals size ranging from 20 to 50 μm, were fabricated using a BioScaffolder 3.1 (GeSiM mbH, Germany). The printed scaffolds were immersed in ultrapurified water (H_2_O) (Arium^®^ pro UF Ultrapure Water System, Germany) for 15 days in order to leach out the sugar particles and create microporosity on the surface and within the scaffold struts. Fabricated scaffolds were sterilized by exposure to 70% ethanol (% v/v) followed by evaporation. Scaffolds were then incubated in 1%HSA and 5%HSA solutions overnight, at room temperature and under agitation. Resulting layers of 1%HSA and 5%HSA were subsequently stabilized/crosslinked by incubating the HSA-coated scaffolds with 10%TA and 1%TA respectively (Supplementary Figure 1).

#### Scanning Electron Microscopy (SEM)

Changes on the morphological microstructure of treated and untreated scaffolds were analyzed by SEM (Tescan MIRA3 FEG-SEM, Australia) at a voltage of 10 kV and a spot size of 12.12. Scaffolds were gold-coated for 75 s at 30 mA by using a Leica EM-SCD005 cool sputter coater 7001F (Leica Microsystems GmbH, Germany).

#### Microcomputed Tomography (μCT)

Printed and leached scaffolds were scanned by microcomputed tomography (μCT40, SCANCO Medical AG, Switzerland) in air at 45 kVp and 177 μA with an isotropic voxel size of 6 μm^3^.

The integration time was set at 300 ms with three times averaging, resulting in a 0.9 s sample time. The scan settings were kept constant for all scanned samples. The images were converted to a DICOM stack for further processing.

#### Porosity and Pore Size Analysis

The microporosity and microscale pore distributions of leached scaffolds were estimated using Avizo 9.5.0 (ThermoFisher Scientific) 3D visualization and analysis software.

The MicroCT scans of three samples were exported as DICOM stacks and then reconstructed in 3D in Avizo. The struts were segmented using the ‘thresholding’ tool. Since the external micropores are connected to the background and both are at the same gray level (representing air), a more complicated approach was required to separate the two. A combination of the ‘grow’ and ‘shrink’ functions was used to create a mask from which the pores were isolated. While this approach is effective in uniform structures, the irregularity of our sample caused some minor segmentation errors which were cleaned up manually. Everything else in the images was designated as background. The final result, using watershed segmentation, was the labeling of three distinct parts – struts, micropores and background.

Based on this segmentation, the microporosity was calculated using the ‘volume fraction’ module. In addition, pores were separated and labeled in 3D using the ‘separate objects’ module. Despite optimization of markers, a small margin of error is expected here (for example in the case of elongated pores) as the module is optimized for convex, near spherical objects. The ‘label analysis’ module was then used to measure the equivalent diameter (Eqdiameter chosen from native measures) of each labeled 3D pore.

#### Mechanical Testing

Mechanical properties of scaffolds were tested by applying uniaxial compression using an Instron model 5848 (Melbourne, Australia) with a 500N load cell. Compression was performed at a rate of 0.1 mm/s, in ddH_2_O at a temperature of 37°C.

#### Fourier Transform Infrared Spectroscopy (FTIR)

Functional groups present on the functionalized scaffolds were determined by collecting the IR absorbance spectra (400–4000 cm^–1^) at a spectral resolution of 1 cm^–1^, by using a Bruker spectrometer (ThermoFisher Scientific, United States).

#### X-ray Photoelectron Spectroscopy (XPS)

Surface chemistry characterization of treated and untreated scaffolds was performed via XPS (AXIS Ultra, Kratos Analytical, United Kingdom). Survey spectra as well as high resolution O 1s, C 1s and N 1s scans were recorded at a pass energy of 150 and 40 eV, respectively. Elements were identified from the survey spectra by using CasaXPS processing software.

#### FITC Labeling of HSA

A solution of 20 mg/ml HSA in 0.1 M sodium bicarbonate buffer (pH 9.0) was mixed with a solution of 20 mg/ml FITC in dimethylsulfoxide at a ratio of 1:100, and let to react overnight. In order to remove unbound FITC, the resulting FITC-labeled HSA solution was transferred into dialysis cellulose tubing with a molecular cut-off of 14,000 Da. After 7 days, purified solution was finally transferred into falcon tubes, freeze-dried and stored at 4°C protected from light.

#### Agar Diffusion Assay

Few colonies of *S. aureus* (ATCC 29213) and *P. aeruginosa* (ATCC 27853) were resuspended and diluted in Mueller-Hinton (MH) broth to a McFarlan unit of 0.8–1.0 at a wavelength of 600 nm, corresponding to a bacteria concentration of 1 × 10^8^ CFU ml^–1^. Resulting inoculum was plated on MH agar plates, untreated and treated scaffolds as well as 6 mm filter disks containing 30 μl of Cefazolin^®^ and Vancomycin^®^ as positive controls against *S. aureus* and *P. aeruginosa*, were subsequently placed and gently pressed onto the agar. Plates were then incubated at 37°C and inhibition zones were measured after 24 h.

#### 3D Broth Assay

*Staphylococcus aureus* (ATCC 29213) colonies were resuspended in MH broth at a concentration of 1 × 10^8^ CFU/mL and placed into 48-well plates (1 mL/well). Treated and untreated scaffolds were placed in each well and incubated at 37°C overnight. Scaffolds with adherent *S. aureus* were washed twice with PBS and subsequently fixed for 3 h with 2.5% glutaraldehyde. Samples were then dehydrated via a series of ethanol treatment by using a Pelco BioWave Microwave Tissue processor. Once dehydrated, the scaffolds were platinum-coated for 75 s at 30 mA by using a Leica EM-SCD005 cool sputter coater 7001F (Leica Microsystems GmbH, Germany). Adhered bacteria to the coated and uncoated scaffolds were then examined under SEM (Tescan MIRA3 FEG-SEM, Australia) at a voltage of 5 kV (spot size 12.12).

#### Statistical Analysis

A minimum of six experimental replicates (*n* ≥ 6, unless otherwise mentioned) were used in each study and the results are presented as mean value ± standard deviation. Data obtained from the *in vitro* antibacterial assays were analyzed using one-way ANOVA (GraphPad Prism 5 software, United States). Differences between the groups were analyzed using the Tukey test of multiple comparisons and a confidence level of 99.9% (*p* < 0.001) was considered as statistically significant, unless otherwise specified.

## Results

### Characterization of Macro- and Microporous Scaffolds

**FIGURE 1:**
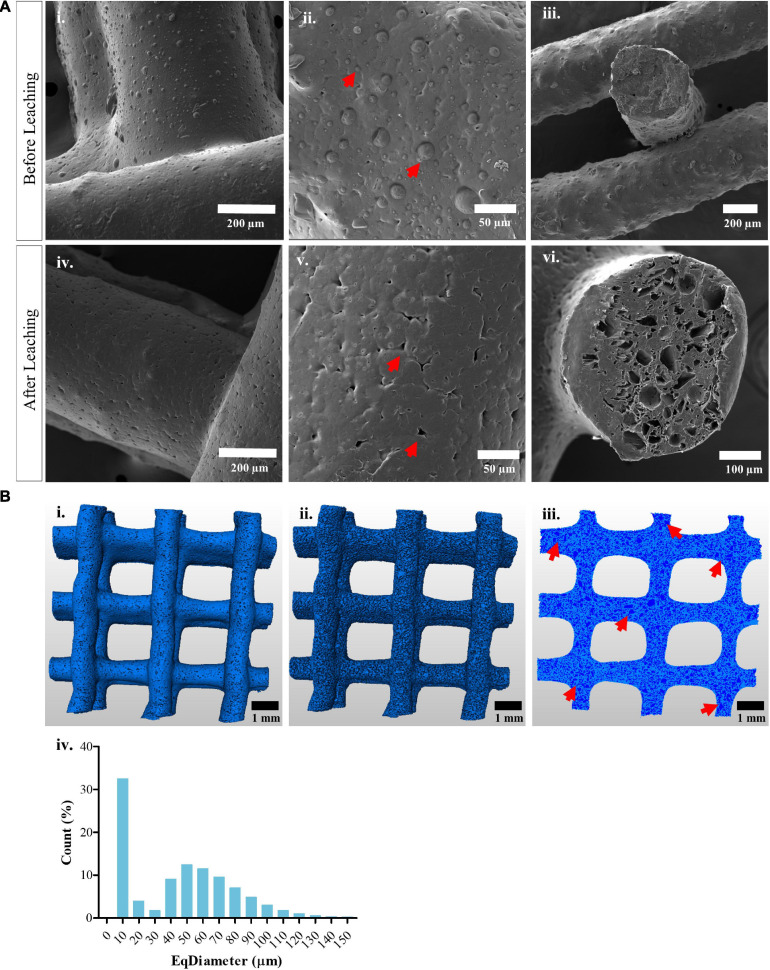
Morphological characterization of macro- and microporous 3D printed scaffolds **(A)** Scanning electron microscopy images showing the surface of extruded mPCL/Sugar scaffolds (i–iii) after printing, red arrows indicate sugar crystals and (iv–vi) after leaching out the sugar particles for 15 days in order to generate micro-sized pores, which are indicated with red arrows. **(B)** μCT evaluation of microporous mPCL scaffolds showing the 3D representation of the (i) segmented scaffold struts after sugar leaching as well as the (ii) segmented micropores. (iii) 2D distribution of micropores across a virtual plane through the scaffold, struts are shown in clear blue and micropores in dark blue, red arrows indicate local interconnectivity of pores. (iv) Equivalent diameter distribution for the micropores present on the surface of and within the scaffolds.

In this study, composite pellets prepared from mPCL and 45% (w/w) of sugar particles with crystal size ranging from 20 to 50 μm, were printed via extrusion in a macroporous lattice structure. Sugar leaching was used to generate micrometer-scale pores on the surface and within the structure of the scaffolds, in order to increase the surface area and facilitate the immobilization of antimicrobial agents on the scaffold surface. SEM imaging of the surface and cross-sections of the fabricated scaffolds confirmed the presence of sugar crystals on the scaffold surface after extrusion (Figure 1Ai–iii), as well as the newly formed micro-sized pores as a result of the sugar-leaching process (Figure 1Aiv–vi). The morphology of the macro- and microporous mPCL scaffolds, including 3D characterization of pore size distribution and interconnectivity, was studied by using high-resolution micro-computed tomography (μCT). The segmentation process resulted in the separation of the scaffold struts (Figure 1Bi) and micropores (Figure 1Bii). Figure 1Biii shows the distribution of microporosity across a plane cut through the scanned sample, evidencing a few locally interconnected pores (red arrows). In order to assess the pores size distribution, the equivalent diameter of each micropore was estimated as the diameter of a sphere with the same volume as each particular micropore. Histogram evaluation of the equivalent diameter distribution of micropores revealed that the isolated pores had an equivalent diameter in the range of 10–150 μm (Figure 1Biv). The presence of micropores with an equivalent diameter greater than 50 μm, is attributed to the agglomeration of sugar particles during the scaffold manufacturing process, which leads to the generation of locally interconnected pores. The total microporosity of the scaffolds was 42.0 ± 0.3%, suggesting that most of the initial sugar content (45%) was leached out from the construct.

### Microporosity Effect on the Mechanical Properties of Clinically Relevant Scaffold Shape and Volume

As a proof of principle toward our group pioneered scaffold-guided breast tissue engineering strategy, we used the above described additive biomanufacturing strategy to 3D print clinically relevant scaffolds (90% porosity with a volume of 75 cm^3^) with a fully interconnected macropore network as well as with micrometer-scale pores on and within the struts. Supplementary Figure 2A shows the scaffolds 3D printed with mPCL and with the mPCL/sugar composite (after leaching). SEM imaging evidenced the presence of small pores on the surface of microporous scaffolds, as a result of the sugar-leaching process, in comparison to the characteristic solid appearance of non-porous mPCL scaffolds (Supplementary Figure 2B). Even though a higher porosity may facilitate cell ingrowth and nutrient transport, the mechanical properties of the scaffold can be affected due to the high amount of void volume within the struts. Therefore, we evaluated the effects of the generated microporosity on the mechanical performance of the scaffolds. Uniaxial compression tests were conducted on both, non-porous and microporous mPCL scaffolds, in water at a temperature of 37°C, to mimic physiological conditions. The force necessary to compress the scaffolds up to 50% strain was 121.5 ± 12.3 N and 75.5 ± 19.8 N for non-porous and microporous mPCL, respectively, evidencing the decreased stiffness of microporous mPCL structures due to the generated microporosity (Supplementary Figure 2D).

### Immobilization of HSA and TA on the Scaffold Surface

**FIGURE 2:**
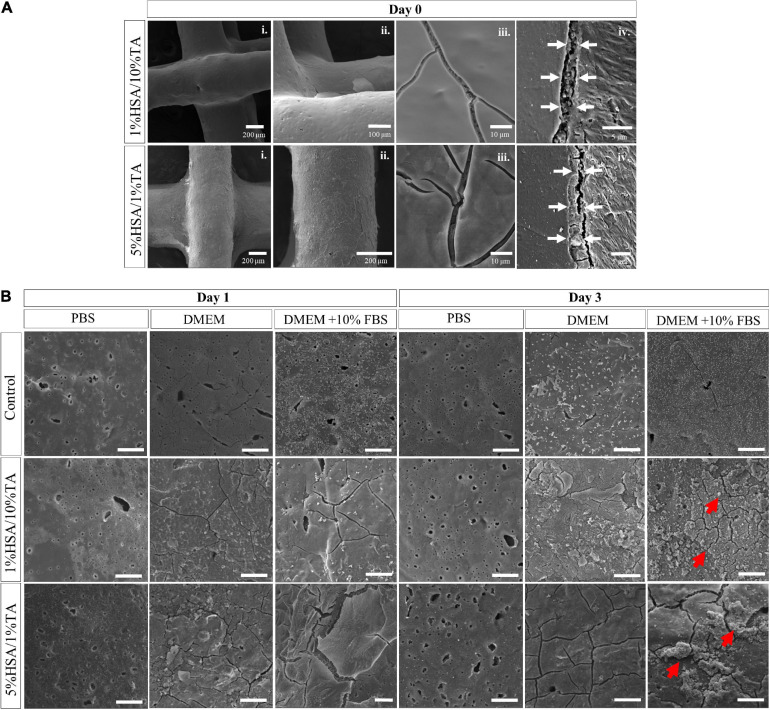
Scanning electron microscopy images showing **(A)** the surface of HSA/TA coated scaffolds after fabrication, from lower (i) to higher (iii) magnifications, as well as (iv) resin-embedded cross sections evidencing distinctive coating thicknesses. **(B)** SEM images of uncoated and coated scaffolds after incubation in PBS, DMEM and DMEM + 10% FBS at 37°C, for 3 days. Red arrows indicate adhered agglomerates on the coated surfaces. Scale bars: 20 μm.

Human serum albumin was first immobilized onto the surface of microporous mPCL scaffolds by physical adsorption and stabilized by treatment with different concentrations of TA. SEM of HSA- and HSA/TA-treated surfaces (Supplementary Figure 2) showed that 0.5%TA stabilization/crosslinking of scaffolds incubated with different concentrations of HSA resulted in a poor immobilization of the antimicrobial agents, as no significant morphological changes were observed on the surface. In contrast, the stabilization of 5%HSA-coated scaffolds with 5 and 10%TA, resulted in a heterogeneous and bulky coating because of HSA precipitation. Therefore, 1%HSA- and 5%HSA-coated scaffolds stabilized with 10%TA and 1%TA respectively, were chosen as possible candidates for the production of highly homogeneous and stable coatings, with the hypothesis that a homogeneous distribution of HSA and TA on the surface would lead to a uniform protection against bacterial colonization throughout the scaffold surface. SEM of scaffolds coated with 1%HSA and 5%HSA and subsequently treated with 10%TA and 1%TA, respectively, evidenced highly uniform thin films covering the entire structures (Figure 2Ai–iii). Small cracks on the coating surface were present as a result of coating dehydration during sample preparation for SEM imaging. Furthermore, cross-sections of resin-embedded scaffolds (Figure 2Aiv) revealed an approximate coating thickness of 2.2 ± 0.2 μm and 3.8 ± 0.2 μm for 1%HSA/10%TA- and 5%HSA/1%TA-treated scaffolds, respectively.

In order to assess the physical stability of both coatings, treated and untreated scaffolds were incubated in PBS, DMEM, and DMEM + FBS for 3 days. SEM analysis was performed to monitor the changes in surface morphology. After 1 day of incubation in the different media, the treated and untreated scaffolds showed new different surface features (Figure 2B). Control scaffolds showed significant adsorption of media components onto the surface when incubated in DMEM and DMEM + FBS, mainly due to the hydrophilicity of microporous scaffolds. Conversely, 1%HSA/10%TA- and 5%HSA/1%TA-coated scaffolds showed little stability when incubated in PBS for 1 day, as both coatings started to wear off from the surface. In contrast, coated scaffolds demonstrated high stability when incubated in DMEM and DMEM + FBS, as the TA-crosslinked HSA film could still be observed in the SEM images after 1 day. After 3 days of incubation in PBS, 1%HSA/10%TA and 5%HSA/1%TA coatings were completely worn off from the surface of the scaffolds. In contrast, both coatings were stable when incubated in DMEM and DMEM + FBS as the surface morphology did not experience significant changes. Particularly, 1%HSA/10%TA and 5%HSA/1%TA coated scaffolds incubated in DMEM + FBS showed several agglomerates of newly adhered compounds from the media. Additional stability studies using FITC-labeled HSA demonstrated that the treated surfaces were highly stable when incubated in DMEM and DMEM + FBS for 3 and 7 days (Supplementary Figure 3). It is hypothesized that the improved stability in DMEM and DMEM + FBS is due to the presence of amino acids and sugars in DMEM, that act as protective osmolytes capable of stabilizing the albumin structure as well as preventing protein aggregation (Jamal et al., 2009; Mojtabavi et al., 2019).

### Surface Characterization

**FIGURE 3:**
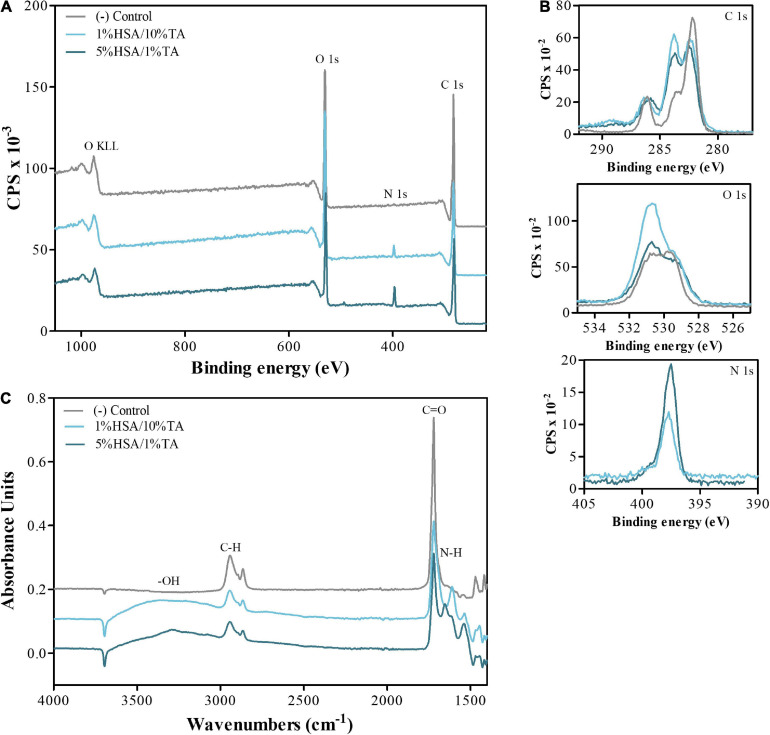
Surface characterization of untreated and treated surfaces. **(A)** Wide and **(B)** high resolution O 1s, C 1s, and N 1s, XPS scan spectra **(C)** FTIR spectra evidencing the presence of hydroxyl and amide groups on the surface of treated scaffolds, demonstrating successful immobilization of HSA and TA on the surface.

X-ray photoelectron spectroscopy (XPS) was used to study the elemental composition of the scaffold surface pre- and post-coating with HSA and TA (Figures 3A,B). As shown in Table 1, blank scaffolds displayed a presence of carbon (%C, 76.0 ± 0.5), oxygen (%O, 23.0 ± 0.3) and a trace of what is presumably silicon contaminate (%Si 1.0 ± 0.2) possibly from lubrication oil used for the moving parts of the 3D printer. In comparison, 1%HSA/10%TA coated scaffolds increased nitrogen (%N) and %O to 3.0 ± 0.3% and 28.3 ± 0.6% respectively. Likewise, 5%HSA/1%TA coated scaffolds also increased %N and %O to 5.8 ± 0.1% and 24.2 ± 0.3% respectively. Changes in the elemental% composition of N and O are indicative of the successful immobilization of HSA and TA on the scaffold surface. Particularly, increase in %O is thought to be mainly contributed by the phenolic hydroxyls of TA and increase in %N is attributed to the characteristic amide groups of HSA.

**TABLE 1:** XPS elemental composition of treated and untreated scaffold surfaces.

Surface	%C	%O	%N	%Si
5%HSA/1%TA	70.0 ± 0.2	24.2 ± 0.3	5.8 ± 0.1	–
1%HSA/10%TA	68.8 ± 0.6	28.3 ± 0.6	3.0 ± 0.3	–
Blank	76.0 ± 0.5	23.0 ± 0.3	–	1.0 ± 0.2

In addition to XPS analysis, Fourier-transform infrared spectroscopy (FTIR) was utilized to identify the functional groups and different types of bonds present on the uncoated and coated scaffolds surface (Figure 3C). Common peaks occurring at 2800–3000 cm^–1^ and 1740–1750 cm^–1^ in the treated and untreated surfaces, are assigned to the vibration of C-H and C=O (ester) groups, which are known characteristic functional groups present on PCL surfaces. In contrast, 1%HSA/10%TA and 5%HSA/1%TA coated surfaces showed particular stretching vibrations at 3200–3550 cm^–1^ and 1580–1650 cm^–1^ corresponding to the presence of –OH and N-H groups, which further confirmed successful immobilization of HSA and TA on the microporous mPCL scaffolds.

### Antibacterial Properties of Scaffolds

**FIGURE 4:**
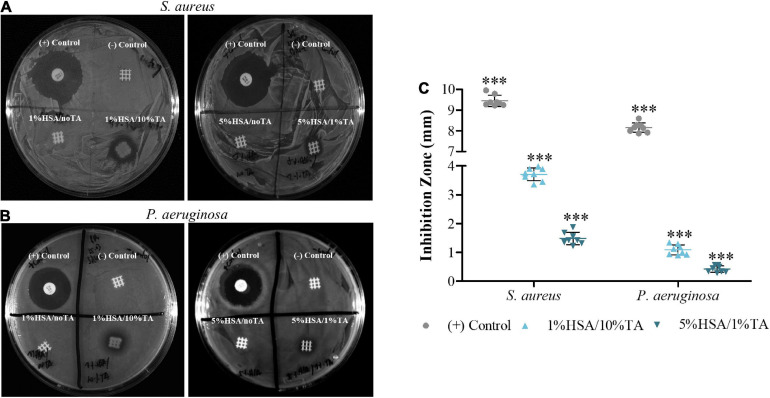
*In vitro* evaluation of treated scaffolds in a 2D zone of inhibition assay against **(A)** gram-positive *S. aureus* and **(B)** gram-negative *P. aeruginosa*. 1%HSA/10%TA- and 5%HSA/1%TA- treated scaffolds exhibited antimicrobial activity against both bacteria strains seen in the form of a clear zone around the samples in the agar plate. **(C)** Comparison of inhibition zones by antibiotic-loaded disks (+Control) and HSA/TA-coated scaffolds. Data shown as mean ± SD, *n* = 8. (^∗∗∗^*p* < 0.001).

Antibacterial *in vitro* efficacy of HSA/TA-coated scaffolds was first assessed in a 2D zone of inhibition assay against *S. aureus* and *P. aeruginosa.* The 1%HSA/10%TA and 5%HSA/1%TA coated surfaces were able to inhibit the growth of both bacteria strains, seen in the form of a clear zone around the samples in the agar plates (Figures 4A,B). Interestingly, HSA-coated scaffolds that were not stabilized with TA did not show any inhibition of *S. aureus* and *P. aeruginosa* growth. Contrastingly, a higher concentration of TA on the surface led to an increase in the inhibition zone (Figure 4C), suggesting that TA contributes to a greater extent to the antibacterial properties of the coatings.

**FIGURE 5:**
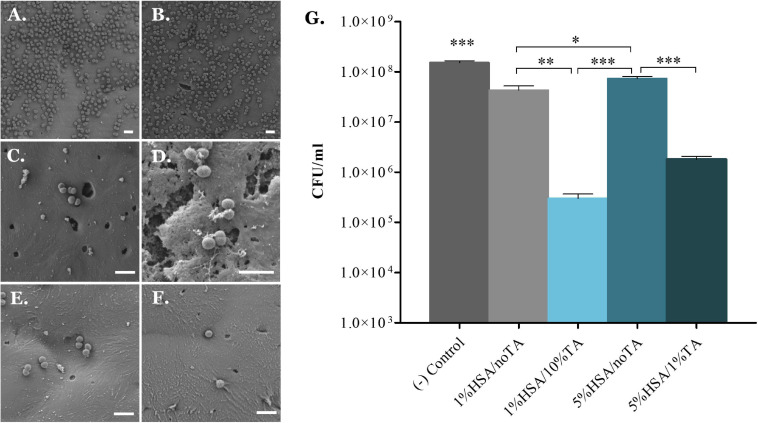
3D *in vitro* evaluation of antibacterial effectiveness of coated scaffolds against *S. aureus* in suspension. **(A,B)** Uncoated scaffolds showed extensive bacterial colonization by *S. aureus*, while **(C,E)** scaffolds coated with 1% and 5%HSA showed significantly fewer adherent bacteria on the surfaces. Scaffolds coated with 1% and 5%HSA and stabilized with 10% and 1%TA, respectively, **(D,F)** did not only evidenced reduced bacteria colonization, but also showed morphological changes on the bacterial membrane suggesting possible membrane disruption. **(G)** Number of viable colony forming units of *S. aureus* recovered from the scaffolds surface. Data shown as mean ± SD, *n* = 6. (^∗^*p* < 0.05, ^∗∗^*p* < 0.01, ^∗∗∗^*p* < 0.001); scale bars: 2 μm.

Since 2D zone of inhibition assays do not fully represent the interactions of coated scaffolds with bacteria in a realistic *in vivo* environment, a 3D *in vitro* assay was designed in order to investigate the antibacterial properties of coated scaffolds against *S. aureus* in suspension. As observed in Figures 5A,B, uncoated surfaces showed extensive bacterial colonization by *S. aureu*s. In contrast, scaffolds coated with 1%HSA and 5%HSA but without TA stabilization reduced *S. aureus* colonization by 72.1 ± 13.6% and 53.5 ± 12.8% (Figures 5C,E), in comparison to the blank group, evidencing the efficacy of HSA for inhibiting bacteria attachment. Interestingly, 1%HSA/10%TA- and 5%HSA/1%TA-treated scaffolds showed a greater reduction of viable CFU (Figure 5G) of 99.8 ± 0.1% and 98.7 ± 0.6%, respectively (Table 2), highlighting the combined antibacterial effects of HSA and TA (Figures 5D,F).

**TABLE 2:** Reduction in *S. aureus* colonization (%) on treated scaffolds.

Surface	Reduction in *S. aureus* colonization (%)
1%HSA/no TA	72.1 ± 13.6
1%HSA/10% TA	99.8 ± 0.1
5%HSA/no TA	53.5 ± 12.8
5%HSA/1% TA	98.7 ± 0.6

## Discussion

Scaffold-guided breast tissue engineering has emerged as a promising alternative to replace conventional post-mastectomy breast reconstruction approaches by using biodegradable polymeric scaffolds that guide tissue regeneration (Hutmacher et al., 2007). In particular, our group has pioneered a unique breast tissue engineering approach based on additive biomanufactured, highly porous, biodegradable mPCL scaffolds in combination with delayed fat injection (Chhaya et al., 2016). Even though this approach allows to achieve sustained regeneration and remodeling of high volumes of soft tissue, there is an intrinsic risk of infection due to persistent bacteria adhering to the biomaterial surface before, during or after implantation (Brooks et al., 2013). Once bacteria adhere to the mPCL scaffold, they rapidly colonize the surface and form biofilms, which are notoriously difficult to treat and can lead to chronic infection and persisting inflammation of surrounding tissue (Jamal et al., 2018). It is therefore vital to ensure the design of mPCL scaffolds that not only support cellular attachment and tissue regeneration, but also prevent bacteria colonization and biofilm formation. Herein, we immobilized HSA and TA, on the surface of 3D printed microporous mPCL scaffolds in order to improve the scaffold biocompatibility, as well as prevent bacteria adhesion and biofilm formation onto the biomaterial surface.

The extraordinary functioning of biological materials, such as breast tissues, is a result of their hierarchical architecture, the specific design at each level of their cellular and extracellular matrix organization. Inspired by these hierarchical structures, as well as by the combination of different biomaterial building blocks and their different properties and environmental requirements, the new field of “materiomics” should converge into the concept of scaffold-guided tissue engineering. Unlike the traditional paradigm in materials science, the connection between the “external” and “internal” properties and requirements of materials take place on different scales that lead to multifunctionality. In addition to multifunctionality, the material requirements are constantly changing (e.g., changing loads, changing biological environment due to tissue regeneration and/or remodeling) on different time and length scales (Ackbarow and Buehler, 2007), therefore, materials for scaffold-guided tissue engineering should be able to address these requirements by having a multifactorial and multifunctional design, not only to replicate the tissue architecture, but to also guide the complex and long-lasting regeneration process.

Based on this background it has been demonstrated that the presence of macro- and micro-scale pores as well as interconnected networks in tissue engineered constructs (TEC), is crucial for tissue ingrowth and vascularization in a preclinical pig animal model (Janzekovic et al., 2020). For instance, interconnected pores with a size greater than 500 μm are required for vascularization in scaffold guided adipose tissue engineering, while micropores ranging from 10 to 50 μm are needed for providing a large surface to volume ratio for increased cell attachment as well as entrapping and stabilizing the initial fibrin network (Murphy and O’Brien, 2010; Oliviero et al., 2012). Notably, macro and micro pore-interconnectivity is a conditio *sine qua non* to facilitate cell migration and diffusion of nutrients (Donnely et al., 2020). The presence of microporosity has also been shown to facilitate the immobilization of different agents or drugs, through physical interactions such as the capillary effect (Dang et al., 2019). In this study, a medical grade PCL composite containing 45% (w/w) of sugar particles with crystal size ranging from 20 to 50 μm, was 3D printed in a macroporous architecture. Leaching of sugar particles in water resulted in a total microporosity of 42.0 ± 0.3%. Interestingly, several newly created micropores showed a diameter greater than 50 μm, suggesting the agglomeration of sugar particles during and after the extrusion process, and resulting in the generation of locally interconnected pores. The increased surface to volume ratio allows greater physical interaction between the antimicrobial agents and the mPCL surface.

Our group has previously published a comprehensive data set, which demonstrated that well designed scaffolds have the ability to regenerate large volumes of the combination of adipose and structured stroma tissue in a porcine model. The force necessary to compress these scaffolds by a 20% strain, decreased from 38.0 ± 1.0 N to 11.4 ± 3.7 N after 24 weeks of implantation, as a result of scaffold degradation and newly formed tissue (Chhaya et al., 2016). Herein, we used FDM and the sugar leaching approach to add microscale porosity to these breast scaffolds and test the effect of the generated microporosity on the mechanical properties of the constructs. The force necessary to reach 20% compression was 52.0 ± 9.4 N for mPCL and 10.1 ± 3.2 N for microporous mPCL, evidencing the reduction in stiffness due to the presence of micropores within the structure. From a biomechanical perspective, scaffolds for large-volume regeneration of adipose tissue, should be flexible and soft to mimic the natural feel and hence biomechanical properties of the breast tissue, while having the sufficient mechanical strength to withstand physiological and external loads to support the regeneration process (Chhaya et al., 2015; Mohseni et al., 2019). Despite the numerous benefits of the incorporation of macro- and micro-scale pores in TECs, porous surfaces have been found to be more susceptible to bacterial colonization as microorganisms harbor themselves in the micropores and evade the host defense system (Merritt and An, 2000). Likewise, the modified mechanical properties due to the incorporation of micropores might lead to cracked or roughened scaffold surfaces that could maximize the contact area between bacterial cells and the surface, further favoring bacteria colonization (Feng et al., 2015). An alternative to reduce the risk of bacterial colonization is to ensure the immobilization of antimicrobial agents throughout the entire microporous surface, as already mentioned. Therefore, the incorporation of macro- and micro-porosity to TECs must be based on a multifactorial design that optimizes the mechanical properties, surface area for retention of biomolecules as well as micropore interconnectivity for cell guidance and tissue ingrowth, while protecting the scaffold surface from bacterial colonization (Hollister, 2005). Future work includes translating of the HSA/TA-coating onto large volume scaffolds to allow mechanical characterization, as well as *in vivo* studies to determine the effectiveness of the coating in promoting implant integration while preventing bacterial infection.

Layer-by-layer assembly of albumin and TA has been widely used for the development of multilayer nanocapsules and hollow multilayer shells which are able to encapsulate, stabilize and deliver different biomolecules such as anticancer drugs (Lomova et al., 2015; Aguilera et al., 2016). Therefore, albumin-TA interactions have been widely studied. For instance, TA interacts with bovine serum albumin (BSA) by forming hydrogen bonding between the amide groups of BSA and the phenolic hydroxyls of TA, as well as by ionic and hydrophobic interactions (Xie et al., 2017). In this study, we used the protein complexing characteristics of TA to immobilize HSA on the surface of microporous mPCL scaffolds. SEM analysis of HSA/TA coated scaffolds as well as confocal microscope and SEM images of scaffolds coated with FITC-labeled HSA, confirmed the coating stability when the samples were incubated in DMEM and DMEM + FBS for 3 days. Both 1%FITC-HSA/10%TA and 5%FITC-HSA/1%TA coatings showed a strong fluorescence signal after 7 days of incubation in DMEM. However, this signal was rather weak when the samples were incubated in DMEM + FBS for the same period. We hypothesize that the FBS molecules present in the media might be interacting with the FITC, causing an interference with the fluorescent signal from the initial FITC-HSA/TA coating. Despite the lack of a strong fluorescent signal, SEM imaging confirmed the presence of both coatings as no significant morphological changes were observed between coated scaffolds incubated in DMEM and DMEM + FBS after 7 days. The improved stability of coated scaffolds in DMEM and DMEM + FBS in comparison to PBS is attributed to amino acids and sugar content in DMEM that can act as stabilizers by preventing protein aggregation as well as inducing refolding of misfolded proteins (Jamal et al., 2009). For instance, Pal et al. (2020) compared BSA stability in sodium phosphate buffer alone and supplemented with glycine, alanine, proline and arginine, amino acids present in DMEM (except for alanine). The authors found that the incorporation of these amino acids to the buffer, allowed BSA to resist unfolding, possibly due to the change in the hydration dynamics of both BSA and the amino acids, confirming the stabilizer-role of glycine, alanine, proline, and arginine (Pal et al., 2020). Nevertheless, no studies on the stability of immobilized albumin when exposed to different buffer compositions have been reported; therefore, further studies are necessary in order to better understand the mechanisms behind the stability of coated scaffolds. Particularly, it is crucial to investigate whether the antimicrobial agents diffuse away from the coating over time. Understanding the release mechanism of these agents as well as determining its effect on the antibacterial properties of the coating over time, are important parameters to optimize the design and fabrication in order to improve the physicochemical stability, biocompatibility and antimicrobial effectiveness of the coating. Alternatives to increase the long-term stability and hence antimicrobial properties of the coated scaffolds include tuning the coating thickness in order to achieve suitable HSA and TA concentrations. This would maximize the antimicrobial action over time while maintaining the concentration threshold of agents immobilized on the surface and released to the surroundings of the implant, thereby avoiding cytotoxicity. In this study, we incubated the microporous mPCL scaffolds in a HSA-rich solution to allow HSA adsorption on the surface overnight; therefore, we hypothesize that longer incubation times could lead to an increase in the coating thickness. Other alternatives to tune the coating thickness include the use of additional coating or surface modification techniques that allow better control over the immobilized doses of antimicrobial agents, such as the use of Layer-by-Layer assemblies, in which multiple layers of HSA and TA can be deposited on the scaffold surface in a controlled-fashion. Nonetheless, this will be the focus of future optimization studies.

Both, HSA and TA have been reported to possess antimicrobial activity against a wide variety of bacteria strains when individually immobilized onto different material surfaces (Kinnari et al., 2005; Liu et al., 2019). However, to the best of our knowledge, no studies have reported the immobilization of these agents on mPCL surfaces, nor the combined antibacterial properties of HSA/TA coatings on any biomaterial surface. Herein, we demonstrate that 1%HSA/10%TA- and 5%HSA/1%TA-coated scaffolds are able to inhibit the growth of *S. aureus* and *P. aeruginosa* in a 2D model. Interestingly, increasing concentrations of TA led to significantly larger inhibition zones in both bacteria strains, whereas HSA showed no effect on bacterial growth. Even though the mechanism by which TA is able to inhibit bacterial growth is not yet fully understood, there is evidence that TA might work as a siderophore, complexing with iron ions and making them unavailable to bacterial cells (Chung et al., 1998; Andrade et al., 2006). Scarceness of iron has an important impact on microorganisms, as it is essential for several metabolic processes involved in bacterial replication, growth and virulence (Doherty, 2007). Other studies suggest that TA might cause bacterial cell wall disruption as a result of TA complexation with enzymes and substrates located on the outer cell membrane (Akiyama et al., 2001; Kaczmarek, 2020). 3D *in vitro* evaluation of the antibacterial effectiveness of coated scaffolds against *S. aureus* in suspension evidenced the synergistic antibacterial effect of HSA and TA. 1%HSA/10%TA- and 5%HSA/1%TA-coated scaffolds did not only reduce bacteria colonization in comparison to scaffolds coated with 1%HSA and 5%HSA but without TA stabilization, but also showed morphological changes on the bacterial membrane, suggesting possible membrane disruption. In agreement with the results obtained in the zone of inhibition assay, a higher concentration of TA led to an improved antimicrobial activity, seen as a reduced bacteria colonization on the scaffold surface. The ability of the HSA/TA coatings to impair bacterial colonization on the biomaterial surface represents a promising strategy to prevent biofilm formation and further local complications such as implant contracture and BIA-ALCL. This is of particular importance as it has been proposed that HSA and TA are less likely to induce survival pressure on bacteria, decreasing the risk of resistance emergence in comparison to antibiotics. However, more studies that investigate in depth the antibacterial mechanism of HSA and TA are needed. Particularly, further *in vitro* characterization of the antimicrobial properties of the newly developed coatings is required to better understand bacterial interactions with the scaffold surface. Future work includes assessing the viability of bacteria adhered to the coated scaffolds. However, this is a challenging task due to TA affinity toward DNA as it has been found that TA competes with DNA-binding dyes used in viability assays, causing a reduction in the emission intensity and hindering the differentiation of viable cells from dead cells (Labieniec and Gabryelak, 2006). Furthermore, it is essential to investigate the capacity of the coatings to maintain the antibacterial properties over longer periods. Biomaterial-related infections usually originate from bacterial contamination during surgery, therefore suitable antimicrobial coatings for clinical applications, should remain reliable and effective during the first days post-operation, preventing both active and dormant microorganisms adhesion on the implant surface (Busscher et al., 2012).

One of the key challenges in the development of antibacterial coatings is to demonstrate both *in vitro* and *in vivo* biocompatibility. Antimicrobial coatings must be able to fulfill their functions under demanding *in vivo* conditions, without having any cytotoxic effect to the host. Additional studies are therefore needed in order to investigate whether the concentrations of HSA and TA used in the coatings are safe for human cells. More importantly, an enhanced understanding of the release mechanism of these components is important as an uncontrolled release might result in cytotoxicity to the surrounding host tissue.

The main limitation of this study is the use of 2D and 3D *in vitro* models that do not fully represent the complexity of the *in vivo* environment. Therefore, future work includes the *in vivo* evaluation of the developed antibacterial surfaces, in order to further investigate the antibacterial properties of the modified surfaces, as well as to assess the effect of HSA and TA on the biocompatibility, with the hypothesis that fibrous encapsulation and capsular contractions are prevented.

## Data Availability Statement

The original contributions presented in the study are included in the article/Supplementary Material, further inquiries can be directed to the corresponding author/s.

## Author Contributions

SC and DH conceived and designed the study. SC conducted all the experiments. SS performed the porosity and pore size analysis. NB, TD, and DH contributed during the evaluation of experiments as well as to the analysis and interpretation of the obtained data. All authors assisted in the preparation and review of the manuscript.

## Conflict of Interest

The authors declare that the research was conducted in the absence of any commercial or financial relationships that could be construed as a potential conflict of interest.
