# Supplementary material for: Antibacterial Albumin-Tannic Acid Coatings for Scaffold-Guided Breast Reconstruction
Source: Front Bioeng Biotechnol. 2021 Mar 31;9:638577. doi: 10.3389/fbioe.2021.638577 (PMC8044405; doi:10.3389/fbioe.2021.638577)
Supplement: Supplementary file 1 [file Data_Sheet_1.docx]

Supplementary Material


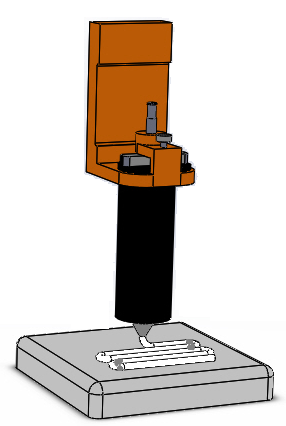

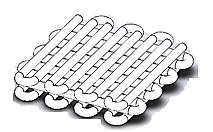

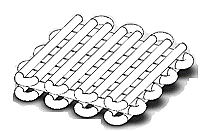


*3D Printing*

*Leaching sugar particles*

*Generated microporosity*

*Albumin coating o/n*

*Stabilizing/crosslinking with tannic acid*

*Coated scaffold*


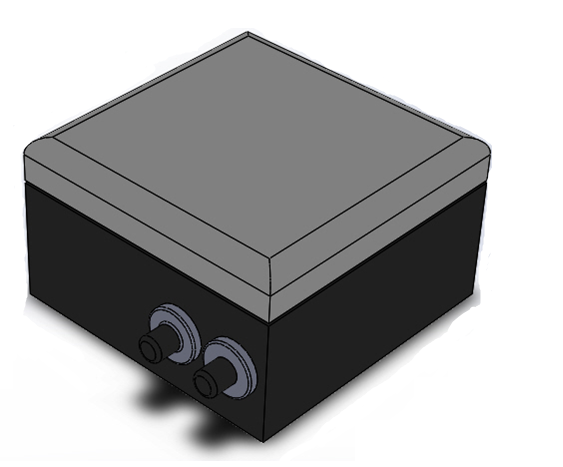

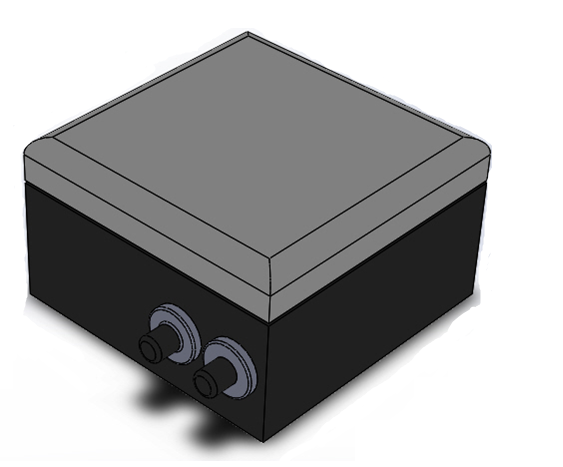

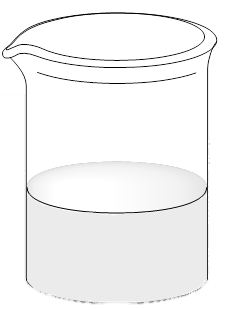

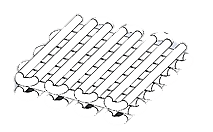

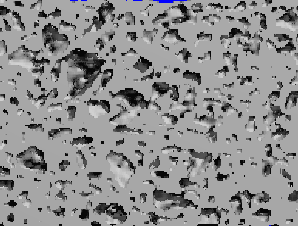

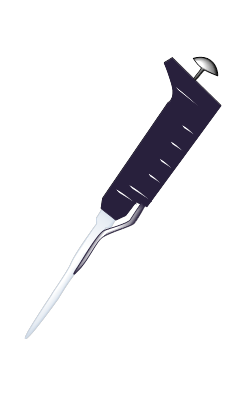

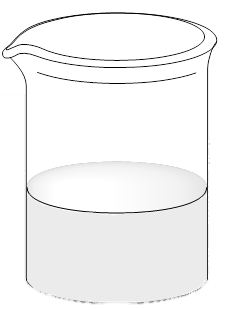

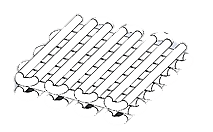

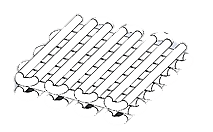


**Supplementary** **Figure 1.** Schematic of additive biomanufacturing of scaffolds. Macroporous mPCL/sugar scaffolds were 3D printed and incubated in H_2_O to leach out the sugar particles and generate a microporosity on the surface and within the scaffolds. Samples were then incubated in HSA overnight under agitation. Resulting deposited HSA on the scaffold surface was subsequently stabilized by incubating the samples in a TA solution.

**C.**


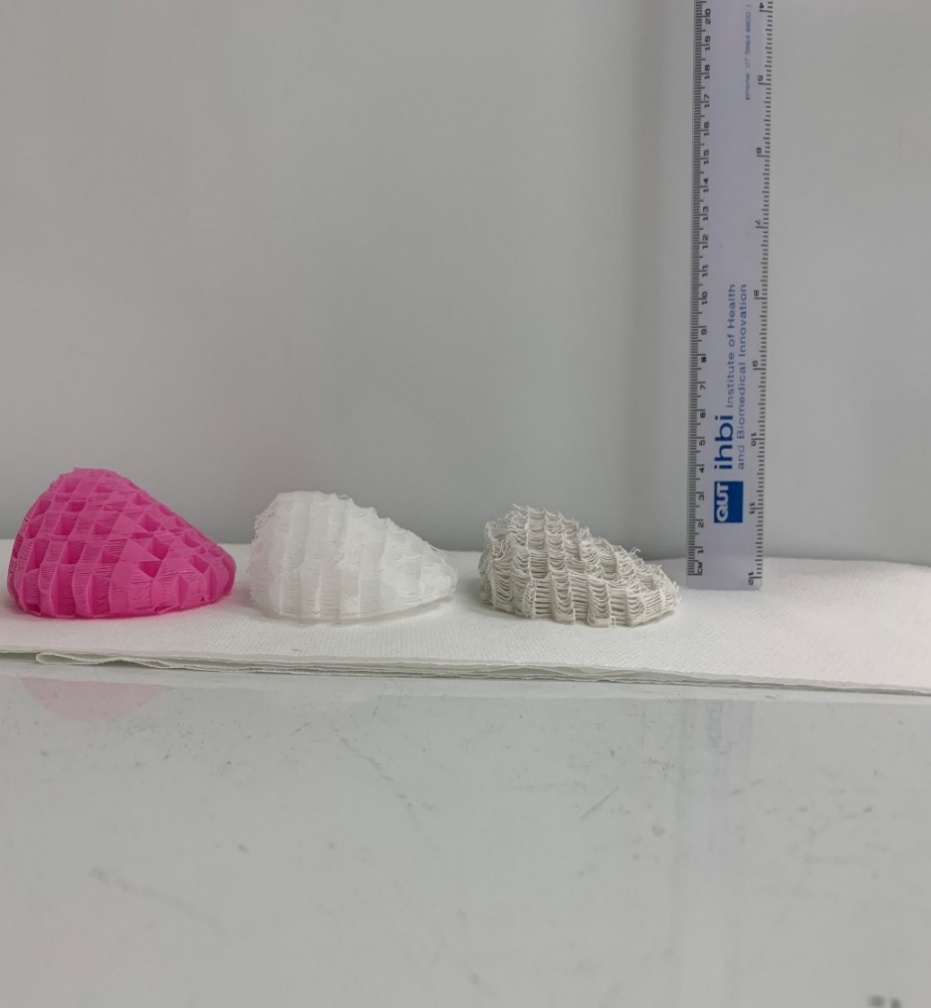

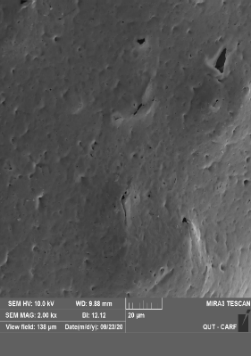

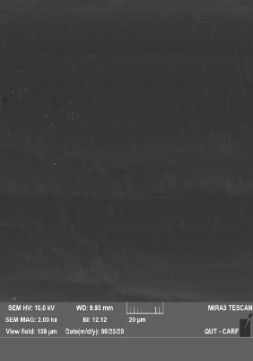


**A.**

**B.**

**D.**


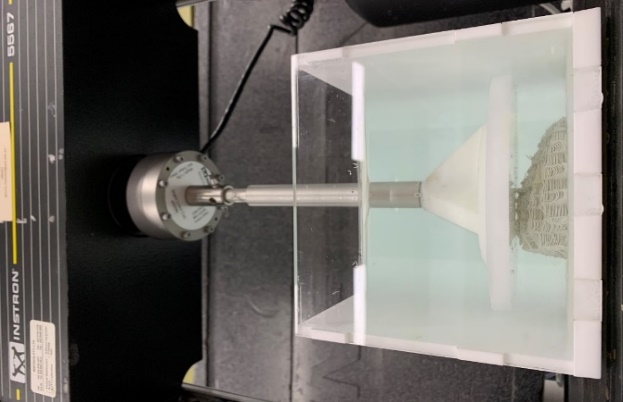


**Load Cell**

**Compression Platen**

**Scaffold**

**Water Tank**

**Supplementary Figure 2.** The study of the biomechanical functionality of tissues through physical contact and stress is a conditio sine qua non. Therefore, to understand the relationship between the structure and functionality of scaffold guided breast reconstruction, it is necessary to start with the quantification of the mechanical behavior of the scaffolds in a simulated physiological condition. (A) Non-porous (left) and microporous (right) clinically relevant sized mPCL scaffolds designed for breast reconstruction. (B) SEM images of non-porous and microporous mPCL scaffolds, red arrows indicated pores generated as a result of the leaching process. Scale bars: 20µm. (C) Uniaxial compression testing set up. (D) Representative force-displacement curves for non-porous mPCL and microporous mPCL scaffolds.

**0% TA**

**0% HSA**


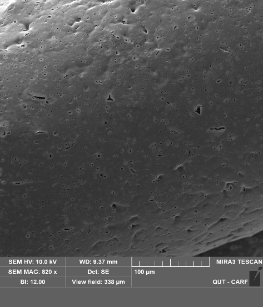

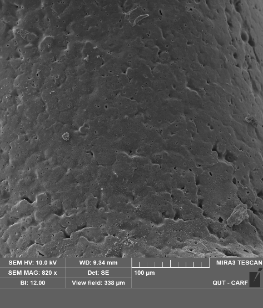

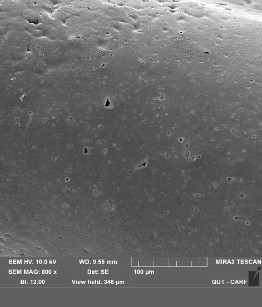

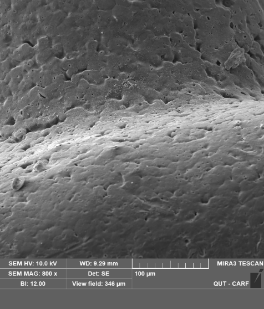

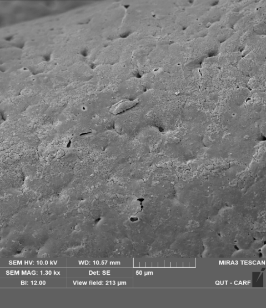

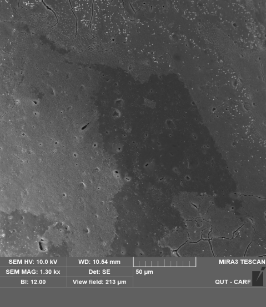

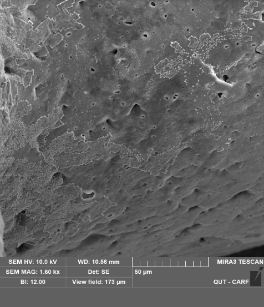

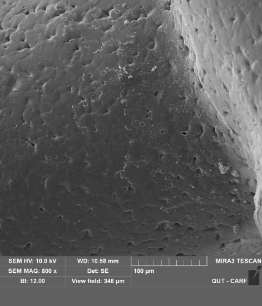

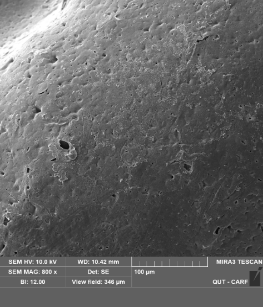

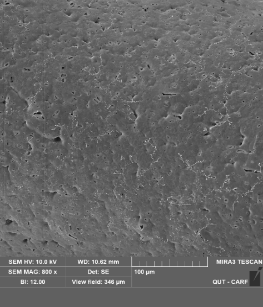

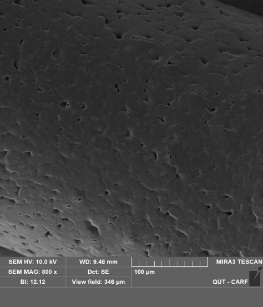

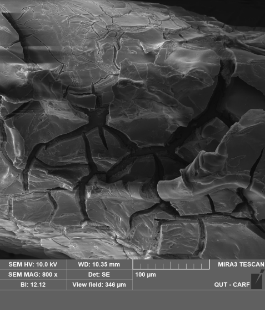

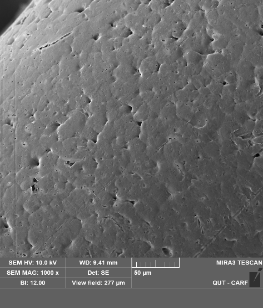

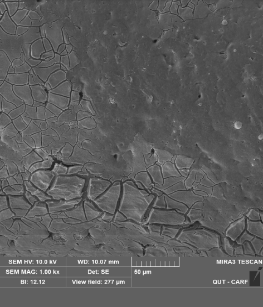

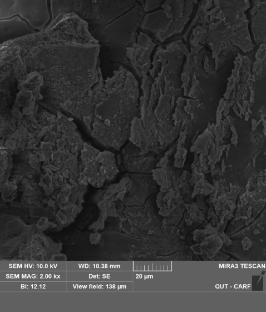


**0% TA**

**0.5% HSA**

**0.5% TA**

**1% TA**

**5% TA**

**10% TA**

**1% HSA**

**5% HSA**

**A.**

**B.**


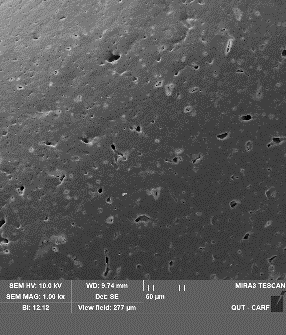

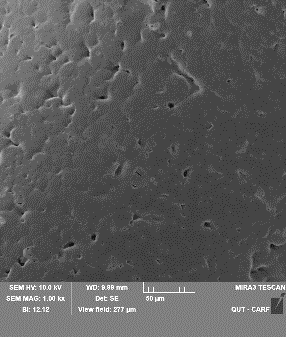


**Supplementary Figure 3.** Scanning electron microscopy images showing the surface of macro- and microporous scaffolds A) untreated as well as B) coated with 0.5%, 1% and 5% HSA and stabilized with 0.5%, 1%, 5% and 10% TA. The presence of TA helped stabilized physically immobilized HSA on the surface of the scaffolds. Scale bars: 50 µm.

PBS

5%HSA/1%TA

**Day 3**

DMEM

DMEM +10% FBS

1%HSA/10%TA

**Day 0**


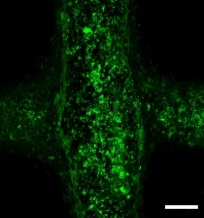

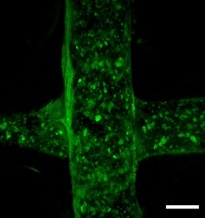

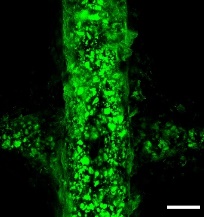

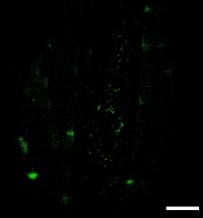

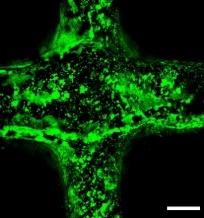

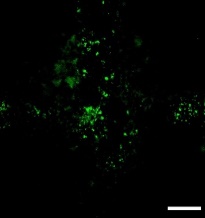

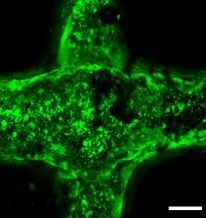

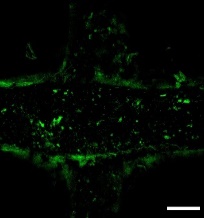

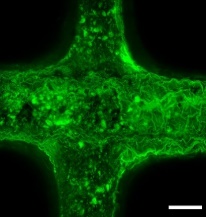

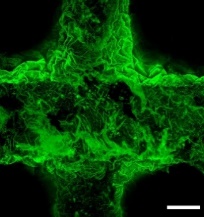

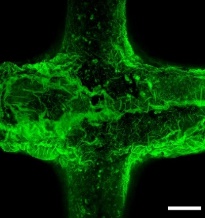


PBS

**Day 7**

DMEM

DMEM +10% FBS


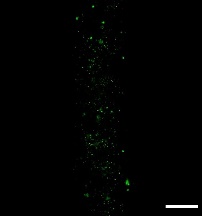

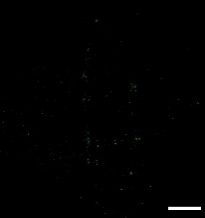

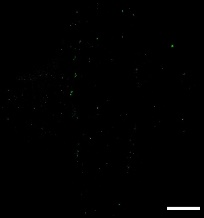


**A.**


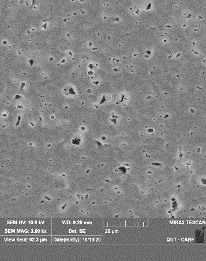

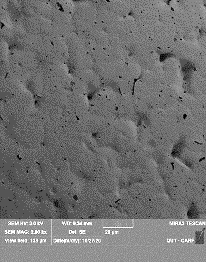

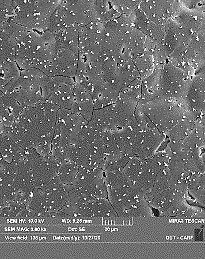

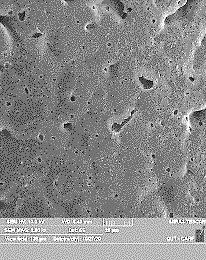

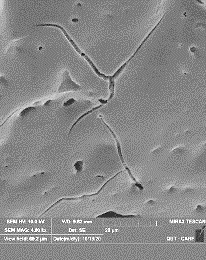

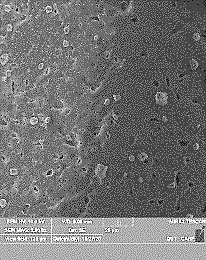

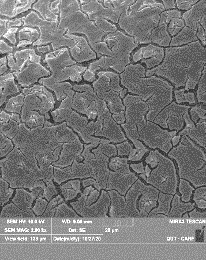

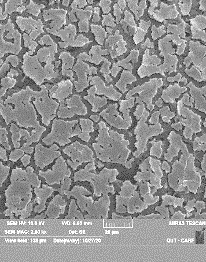

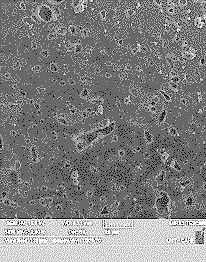

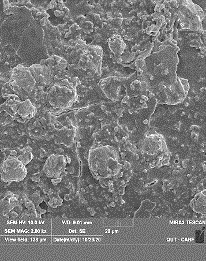

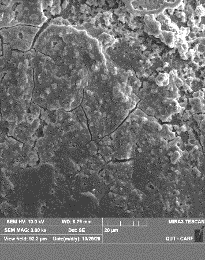

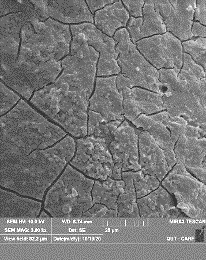

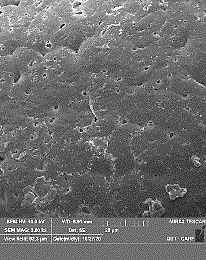

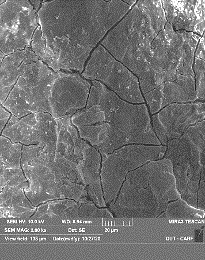

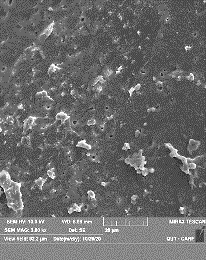

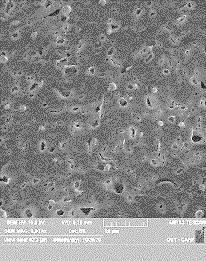

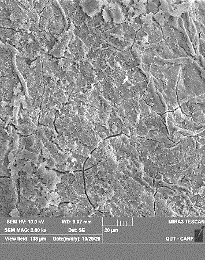

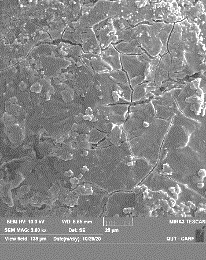

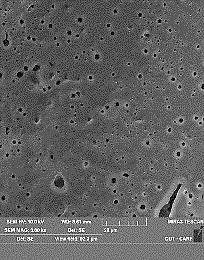

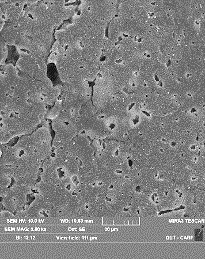

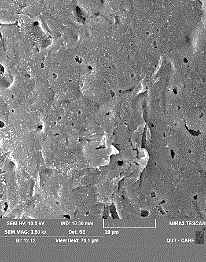


PBS

5%HSA/1%TA

Control

**Day 3**

DMEM

DMEM +10% FBS

1%HSA/10%TA

**Day 7**

PBS

DMEM

DMEM +10% FBS

**Day 0**

**B.**

Supplementary Figure 4. Stability of HSA/TA coatings after 7 days of incubation in different buffer solutions at 37°C. (A) Confocal microscope and (B) SEM images of scaffolds coated with 1% and 5% FITC-labelled HSA and stabilized with 10% and 1% TA, respectively. FITC-HSA/TA-coated scaffolds incubated in PBS showed little stability, while coated scaffolds incubated in DMEM and DMEM+FBS were shown stable over 7 days. Scale bars: 250 µm (A) and 20 µm (B).
